# Improved Wavelengths and Energy Levels of Doubly-Ionized Argon (Ar III)

**DOI:** 10.6028/jres.101.067

**Published:** 1996

**Authors:** Victor Kaufman, Ward Whaling

**Affiliations:** National Institute of Standards and Technology, Gaithersburg, MD 20899-0001; California Institute of Technology, Pasadena, CA 91125

**Keywords:** argon, energy levels, parametric fit, spectrum, wavelengths, wavenumbers

## Abstract

New measurements of Ar III wavelengths between 508 Å and 4183 Å are combined with measurements from the literature to find improved values for the energy of most of the known levels in Ar III. Parameters derived from fitting the new level energies to an *LS*-coupling model are presented along with eigenvector compositions of the levels. On the basis of this analysis new designations are recommended for several levels.

## 1. Introduction

The energy levels of Ar III in Vol. 1 of *Atomic Energy Levels* [1] (1948) are based on de Bruin’s 1937 paper [2]. A complete list of publications pertaining to observed wavelengths and energy levels published prior to 1948 is contained in Ref. [1]. de Bruin reported only the three lowest singlet terms (3*s*^2^ 3*p*^4 1^D and ^1^S, and 3*s* 3*p*^5 1^P^o^), and the work published since 1948 has been concerned primarily with identifying singlet terms at higher energy. Fawcett et al. [3], in 1968, identified three lines at 389 Å, 387 Å, and 382 Å as transitions to 3*s*^2^ 3*p*^4 1^D_2_ from 3*s*^2^ 3*p*^3^(^2^D) 4*d* ^1^P_1_, ^1^D_2_, and ^1^F_3_, respectively. In 1975 Marling [4] identified three singlet-singlet transitions between levels of 3*s*^2^ 3*p*^3^ 4*p* and 3*s*^2^ 3*p*^3^(3*d* and 4*s*). Agentoft et al. [5] confirmed in 1984 an identification of the 3*s*^0^ 3*p*^6 1^S_0_ by McGuire [6].

In 1987 Hansen and Persson [7] published a revised analysis of the Ar III spectrum that contained all possible levels of the 3*s*^2^ 3*p*^4^, 3*s* 3*p*^5^, 3*s*^0^ 3*p*^6^, 3*s*^2^ 3*p*^3^ (4*s*, 4*p*, and 3*d*) configurations. However, they include no observational data in their paper, and their energy values were given to only one decimal place. A recent paper by Whaling et al. [8], primarily concerned with the Ar I and Ar II spectra, identified about 60 lines of Ar III in the near ultraviolet. In the present paper we present improved level energies in Ar III derived from the best wavelengths in the literature, from new wavelengths measured expressly for this work, and from some older but hitherto unpublished wavelengths. A calculation of the Ar III level system in terms of *LS*-coupling suggests that several levels should be renamed.

## 2. Spectra

Wavelengths longer than 2250 Å were measured on 13 spectra from the archives of the National Solar Observatory (NSO); all were recorded on the 1 m Fourier Transform Spectrometer (FTS). Wavelengths between 2800 Å and 1850 Å were measured on spectra from the vacuum FTS at Lund University, Lund, Sweden. All spectra measured on the FTS were excited in an Ar discharge in a metal hollow-cathode source operating 0.13 kPa to 0.53 kPa (1 Torr to 4 Torr) and 0.1 kW to 1.0 kW in a cathode cavity 3 mm in diameter and 25 mm long. Several different cathode metals were used, and spectra were also recorded with Ne replacing the Ar to aid in the identification of Ar lines in the crowded metal spectrum. Further details of the spectral source will be found in Ref. [8].

Wavelengths shorter than 1850 Å were measured on spectrograms recorded at the National Bureau of Standards (NBS), now NIST, in 1971. A pulsed-rf source was used with halides of Na, Li, Ge, and Si during the study [9] primarily aimed at producing the spectra of singly and doubly ionized chlorine (Cl II and III). Argon was used to assist the discharge. The spectra were recorded photographically using the 10.7 m normal incidence vacuum spectrograph. Lines of C, N, O, Si, Ge, and Cu [10] were used as reference wavelengths for the reduction of the spectrograms by polynomial interpolation.

For all FTS spectra the wavenumber scale was calibrated using internal Ar II wavenumber standards derived from heterodyne measurements of CO molecular bands [11] as described in Ref. [8]. All FTS spectra were measured with the DECOMP [12] analysis program developed at the NSO by J. W. Brault. This program fits a Voigt profile to the observed feature and records the line-center wavenumber, peak amplitude, *FWHM*, and other parameters of the Voigt profile. The observed line-center wavenumber in vacuum has been converted to an observed air-wavelength (for *λ*> 2000 Å) using the expression for the index of refraction developed by Peck and Reeder [13] and appears in the first column of Table 1.

As a rough indication of the relative intensity of the Ar III lines as produced in the hollow cathode source, we list in column 2 of Table 1 the logarithm of the ratio (line amplitude)/(rms noise level) for lines measured on FTS spectra. The amplitude has not been corrected for the response of the spectrometer, and a comparison of two line amplitudes is meaningful only for neighboring lines. We are unable, of course, to include the intensity of lines in Table 1 not seen in our spectra, nor do we know the intensity of the far VUV lines measured at the NIST.

We have incorporated in our linelist a few additional wavelengths from the literature that establish important links between levels or that enable us to find values for levels that do not appear in our spectra. Five magnetic dipole lines measured by Bowen [14a,14b] in astronomical spectra, and a measurement in a planetary nebula by Kelly and Lacy [15], determine the spacing between several levels of the ground configuration; we have included their results in the least-squares fit of the levels to the lines on an equal footing with our own. de Bruin [2] has published values for several transitions that join the levels we have measured with levels in the 3*d*″ ^3^D, 3*d*′ ^3^S, 3*d*′ ^3^P, and 4*d*′ ^3^S terms. We have included de Bruin’s measurements in Table 1 (identified with the notation D) and used them to find the energy of these levels.

It is important to establish the uncertainty of the measured wavelengths because the uncertainties play an important role in the weighted least-squares analysis that we used to extract level energies from the measured transition energies. Of the several factors that limit the accuracy of interferometric wavenumber measurements, the important ones for the FTS spectra we used are the line-width produced in the hollow-cathode, and the noise continuum of broad-range FTS spectra. For a line well-separated from its nearest neighbor, we assign a standard uncertainty (i.e., 1 standard deviation estimate) to the measured line position of *δWN* = 0.5(*FWHM*)/(*S*/*N*), where the full width at half maximum *FWHM*, the peak amplitude *S*, and the RMS background noise level *N*, are parameters generated by the DECOMP line-fitting program. The factor 0.5 is a convenient approximation to the more precise, but difficult to evaluate, expression given by Brault [12]. For very strong lines (*S*/*N* ≥ 10^3^) the expression above may underestimate *δWN*, as discussed in Ref. [8], and we therefore set a lower limit *δWN*_min_ = 0.001 cm^−1^. *δWN* varies from line to line and increases with wavenumber because of the increasing Doppler width; it is typically less than 0.010 cm^−1^.

For lines measured with the grating spectrometer the standard uncertainty in wavelength *δWL*_VUV_ should be the same for all wavelengths, and we have set *δWL*_VUV_ = ± 0.001 Å by analyzing the internal consistency of the network of 209 interconnected transitions used in the weighted least-squares analysis. Starting with a conservative estimate of *δWL*_VUV_ = ± 0.004 Å, and giving each observed transition energy *WN*_Obs_ the weight *w_i_* = (*δWN_i_*)^−2^, we found that the residual Σ(*w_i_*×(*WN*_Calc_ − *WN*_Obs_)^2^)/Σ*w_i_* was determined by *internal* consistency alone. Only when the wavelength uncertainty was reduced below 0.0009 Å did the residual show a significant increase, and we have set *δWL*_VUV_ = ± 0.001 Å for all lines measured with the grating spectrometer.

For the wavelengths of the magnetic-dipole transitions taken from the literature we have adopted the uncertainty estimates of the original authors. The wavelengths measured by de Bruin [2] have been assigned a standard uncertainty of 0.02 Å, a value suggested by a comparison of his values with those measured on FTS spectra.

## 3. Line Identification

The hollow-cathode spectra contain Ar I, II, and III lines, plus lines from two or more stages of ionization of the cathode element. An Argon linelist was extracted from this mixture by finding lines common to spectra from two or more cathodes. Ar I and II lines, identified using the linelist published in Ref. [8], were removed and the remaining Ar lines were sufficiently sparse that the rough Ar III wavelengths compiled in Refs. [16] could be used to classify many of the remaining lines. In addition, we used transition energies computed from the Ar III level energies of Hansen and Persson [7] to identify singlet-singlet transitions not included in Refs. [16]. This initial list of classified Ar III transitions was further refined after we had obtained precise Ar III level energies and were able to calculate accurate transition energies and search our observed spectra with a small (±0.010 cm^−1^) search window.

## 4. Level Energies

Level energies were derived from our measured transition energies with the CLEVEL least-squares code of Palmer and Engleman [17] that solves a set of overdetermined linear equations (of the form *E_b_* − *E_a_* = *WN_ba_*) for the most probable energy values and their standard uncertainties, *E_i_* ± *δE_i_*. It is necessary to pay careful attention to the uncertainty assigned to each measurement.

The Ar III level energy values and their uncertainties, *E_i_* ± *δE_i_* produced by CLEVEL are listed in Table 2. For each input line, CLEVEL evaluates the *calculated* value of the transition energy from *WN_ba_*_,Calc_ = *E_b_* − *E_a_* (see column 2 of Table 1) and the standard uncertainty of this calculated wavenumber which appears in parentheses following *WN*_Calc_. We list also the difference *WN*_Obs_ − *WN*_Calc_ in column 3 of Table 1.

Note that the standard uncertainty of the calculated wavenumber (from the appropriate element of the covariant matrix) is often very much smaller than the standard uncertainty of either level involved in the transition with respect to various other levels, including the ground level. These small uncertainties go with transitions between high levels of the same multiplicity and core configuration, and the difference in level energies depends only on the *relative* energy of the two levels and is independent of VUV transitions to the ground term. Any other transition between two such levels should likewise have a small standard uncertainty, and for this reason we have listed the level energies in Table 2 with more decimal places than appears to be justified by the standard uncertainty of the *absolute* energy of a level. For a calculated transition energy between dissimilar levels that are tied together only by transitions down to the ground term and back up again (e.g., between a quintet level and a triplet level), the standard uncertainty is much larger. For dissimilar levels the standard uncertainty should be estimated by combining in quadrature the uncertainty listed in Table 2 for the absolute energy of the initial and final level of the transition.

## 5. Theory

The ground state of doubly-ionized argon has the electron configuration 1*s*^2^ 2*s*^2^ 2*p*^6^ 3*s*^2^ 3*p*^4^, which gives rise to ^3^P, ^1^D, and ^1^S terms. All of the observed excited configurations (except 3*s* 3*p*^5^ and 3*p*^6^) result from the excitation of a 3*p* electron into a higher orbital to form 3*p*^3^ *nl* configurations. The parent configuration, 3*p*^3^, of Ar IV forms the terms ^4^S^o^, ^2^D^o^, and ^2^P^o^. The arrangement of levels in all of the 3*p*^3^ *nl* configurations given herein (except 3*p*^3^ 3*d*) is dominated by the separation of the parent terms.

In Table 3 we give the parameter set derived from a least-squares fit of the levels of the 3*s* 3*p*^5^, 3*s*^2^ 3*p*^3^ (3*d* and 4*d*) configurations with the HFR Cowan code [18]. Also included are the HFR (relativistic approximations to the Hartree-Fock) values obtained and the ratio of the fitted value to that of the HFR value. Table 4 gives the eigenvector composition in the *LS*-coupling scheme of all of the levels of these three configurations including those for which we were unable to obtain experimental values. It is evident from the table that the 3*p*^3^ 4*d* configuration is only very slightly perturbed by the 3*s* 3*p*^5^ and 3*p*^3^ 3*d* configurations; the coupling appears to be very close to LS. There is very strong parental mixing among the three ^3^D terms of the 3*p*^3^ 3*d* configuration, as was seen by Hansen and Persson [7]. Because of the difference between their parametric fit and ours, we have labeled the ^3^D terms near 156 900 and 187 800 cm^−1^ as having the ^4^S and ^2^D parents, respectively, while they did the reverse.

It should be noted that Hansen and Persson [7] did a parametric fit to the 3*s* 3*p*^5^ + 3*s*^2^ 3*p*^3^ (3*d* + 4*s*) configurations. On the basis of that fit, they named the terms at about 189 000 cm^−1^ and 214 000 cm^−1^ as 3*d*″ ^3^P and 3*d*′ ^3^P, respectively, in agreement with our designation for these terms. However, they found such strong mixing of the 3*p*^3^ (^2^D) 3*d* ^3^P and the 3*p*^3^ (^2^P) 4*s* ^3^P eigenvectors in the term at 214 000 cm^−1^ that it may be misleading to label this term with any specific core designation. Thus, the term at 214 000 cm^−1^, although labeled 3*d*′ ^3^P implying a ^2^D core, takes part in 17 known optical transitions, and, in all 17 cases, the transition partner has a well-established ^2^P core; no transition to a level with a ^2^D core has been reported.

It was shown that in Cl II [9] the lowest odd ^1^P_1_ level had only 33 % of 3*s* 3*p*^5 1^P and 57 % of 3*p*^3^(^2^D) 3*d* ^1^P_1_ and that level was given the latter designation. As shown in Table 4, the level at 144 022 cm^−1^ has only 43 % of 3*s* 3*p*^5 1^P and 50 % of 3*s*^2^ 3*p*^3^(^2^D) 3*d* ^1^P. However, because the next higher ^1^P level at 219 908 cm^−1^ also has a larger eigenvector percentage of ^1^P from the (^2^D) 3*d* configuration, we have designated the lower level as 3*s* 3*p*^5 1^P.

Hansen and Persson [7] have given calculations for the 3*p*^4^ and the 3*p*^3^ 4*p* configurations. With only minor exceptions, they find almost no interaction between levels of different parentage in the 3*p*^3^ 4*p* configuration. They find, however, relatively large configuration interaction affecting the ^1^S_0_ levels of the 3*p*^4^, 3*p*^3^ 4*p*, and 3*p*^6^ configurations.

## 6. Ionization Energy

In their paper on ionization energies of singly ionized rare earths, Sugar and Reader [19] showed that it is possible to use the difference in effective quantum numbers of unperturbed adjacent members of an *ns* series to calculate an ionization energy. Using published values of the ionization energies and 3*p^N^* 4*s* and 3*p^N^* 5*s* levels of Ca III and Sc III, we arrive at an average of *δn* = 1.0312. With this value and the experimentally determined value of *δT* = *E*(5*s* ^5^S_2_) − *E*(4*s* ^5^S_2_) = 76 341.079 cm^−1^ in Ar III, the equation

$$\delta T=\frac{Z^{2}R}{{\left[n\left(4s\right)\right]}^{2}}-\frac{Z^{2}R}{{\left[n\left(4s\right)+n\right]}^{2}}$$

where *Z* = 3, was solved for *n*(4*s*). The corresponding value of *T*(4*s*) = *Z*^2^*R*/[*n*(4*s*)]^2^ when added to the level value of 4*s* ^5^S_2_, gives the ionization energy as 328 550 cm^−1^. Although the values of *δn* for both Ca III and Sc III are in near agreement and would therefore lead to a relatively small value for the uncertainty of the calculated ionization energy, we feel that with such a small sampling of possible values of *n* it is necessary to place a standard uncertainty of approximately ± 100 cm^−1^ on the ionization energy. The value of 328 550 ± 100 cm^−1^ compares very well with the value of 328 600 cm^−1^ obtained by Edlén [20] using a semi-empirical isoionic method.

## Figures and Tables

**Table 1:** Classified Ar III lines used for determining the level energies given in Table 2. Observed wavelength in the first column is in air for 2000 Å < *λ* < 10 000 Å; otherwise in vacuum. Calculated vacuum wavenumber in the second column is followed by its standard uncertainty in parentheses. The third column displays the difference between the observed (O) and calculated (C) wavenumber; the value 0.000 means < 0.0005. The meanings of the designations KL, B, D, B2, and * are given at the end of the table

Wavelength (Å)	Vacuum wavenumber and standard uncertainty (cm^−1^)	O–C (cm^−1^)	Classification	
89 913.8 KL	1 112.176 (0.015)	0.001	3*p*^4 3^P_2_	3*p*^4 3^P_1_
7 751.06 B	12 897.787 (0.046)	0.12	3*p*^4 3^P_1_	3*p*^4 1^D_2_
7 135.80 B	14 010.009 (0.045)	−0.03	3*p*^4 3^P_2_	3*p*^4 1^D_2_
5 191.82 B	19 255.72 (0.24)	−0.01	3*p*^4 1^D_2_	3*p*^4 1^S_0_
4 182.9667	23 899.742 (0.001)	−0.000	4*s*′ ^1^D_2_^o^	4*p*′ ^1^P_1_
4 088.8900	24 449.603 (0.002)	0.002	3*d*′ ^1^P_1_^o^	4*p*″ ^1^D_2_
4 059.89 D	24 624.31 (0.11)	−0.05	3*d*′ ^3^P_0_^o^	4*p*″ ^3^S_1_
4 023.5864	24 846.439 (0.005)	−0.012	3*d*′ ^3^P_1_^o^	4*p*″ ^3^S_1_
3 960.4873	25 242.272 (0.003)	0.004	3*d*′ ^3^P_2_^o^	4*p*″ ^3^S_1_
3 907.84 D	25 582.28 (0.11)	0.06	3*d*′ ^3^P_0_^o^	4*p*″ ^3^D_1_
3 858.2923	25 910.853 (0.002)	0.002	3*d*′ ^3^P_1_^o^	4*p*″ ^3^D_2_
3 795.3435	26 340.600 (0.002)	−0.003	3*d*′ ^3^P_2_^o^	4*p*″ ^3^D_3_
3 637.8731	27 480.757 (0.001)	−0.000	4*s*′ ^1^D_2_^o^	4*p*′ ^1^F_3_
3 514.2005	28 447.837 (0.002)	0.002	4*s* ^3^S_1_^o^	4*P*^3^P_1_
3 511.6671	28 468.364 (0.002)	−0.002	4*s*′ ^3^D_3_^o^	4*p*′ ^3^D_2_
3 511.1485	28 472.568 (0.002)	−0.002	4*s* ^3^S_1_^o^	4*p* ^3^P_2_
3 509.3334	28 487.293 (0.002)	−0.000	4*s* ^3^S_1_^o^	4*p* ^3^P_0_
3 503.5892	28 533.995 (0.002)	0.001	4*s*′ ^3^D_2_^o^	4*p*′ ^3^D_2_
3 502.6829	28 541.376 (0.002)	0.003	4*s*′ ^3^D_2_^o^	4*p*′ ^3^D_1_
3 499.6693	28 565.955 (0.002)	0.001	4*s*′ ^3^D_1_^o^	4*p*′ ^3^D_1_
3 497.10 D	28 586.97 (0.13)	−0.03	3*d*″ ^3^D_1_^o^	4*p*″ ^3^D_1_
3 484.12 D	28 693.42 (0.13)	0.02	3*d*″ ^3^D_1_^o^	4*p*″ ^3^D_2_
3 480.5022	28 723.267 (0.002)	−0.003	4*s*′ ^3^D_3_^o^	4*p*′ ^3^D_3_
3 417.49 D	29 252.63 (0.12)	0.22	3*d*″ ^3^D_2_^o^	4*p*″ ^3^D_2_
3 413.53 D	29 286.55 (0.12)	0.25	3*d*″ ^3^D_2_^o^	4*p*″ ^3^D_3_
3 391.8445	29 474.023 (0.002)	−0.000	3*d*″ ^3^P_2_^o^	4*p*″ ^3^P_2_
3 358.5305	29 766.375 (0.002)	−0.002	4*s*′ ^3^D_1_^o^	4*p*′ ^3^F_2_
3 344.7566	29 888.948 (0.002)	0.001	4*s*′ ^3^D_2_^o^	4*p*′ ^3^F_3_
3 342.5373	29 908.793 (0.001)	−0.000	3*d*′ ^1^P_1_^o^	3*p*^6 1^ S_0_
3 336.1746	29 965.832 (0.002)	0.001	4*s*′ ^3^D_3_^o^	4*p*′ ^3^F_4_
3 327.34 D	30 045.38 (0.14)	0.01	3*d*″ ^3^D_3_^o^	4*p*″ ^3^D_2_
3 323.59 D	30 079.29 (0.14)	0.001	3*d*″ ^3^D_3_^o^	4*p*″ ^3^D_3_
3 311.2423	30 191.453 (0.003)	0.003	4*s* ^5^S_2_^o^	4*p* ^5^ P_1_
3 301.8546	30 277.290 (0.002)	0.003	4*s* ^5^S_2_^o^	4*p* ^5^ P_2_
3 285.8413	30 424.844 (0.002)	−0.003	4*s* ^5^S_2_^o^	4*p* ^5^ P_3_
3 251.7907	30 743.419 (0.001)	−0.000	4*s*″ ^1^P_1_^o^	4*p*″ ^1^ P_1_
3 187.90 D	31 359.53 (0.13)	0.01	3*d*″ ^3^D_1_^o^	4*p*″ ^3^P_0_
3 110.41 D	32 141.23 (0.12)	−0.45	3*d*″ ^3^D_2_^o^	4*p*″ ^3^P_1_
3 109.08 B2	32 153.51 (0.24)	1.02	3*p*^4 3^P^1^	3*p*^4 1^ S_0_
3 083.64 D	32 419.97 (0.12)	−0.18	3*d*″ ^3^D_2_^o^	4*p*″ ^3^P_2_
3 054.7736	32 726.134 (0.004)	−0.000	4*s*″ ^3^P_1_^o^	4*p*″ ^3^D_2_
3 023.9801	33 059.372 (0.006)	0.002	4*s*″ ^3^P_2_^o^	4*p*″ ^3^D_3_
3 010.02 D	33 212.71 (0.14)	−0.02	3*d*″ ^3^D_3_^o^	4*p*″ ^3^P_2_
3 002.6408	33 294.312 (0.002)	−0.000	4*s*″ ^1^P_1_^o^	4*p*″ ^1^ D_2_
2 884.2142	34 661.318 (0.002)	0.004	4*s*′ ^3^D_3_^o^	4*p*′ ^3^P_2_
2 878.7636	34 726.950 (0.002)	−0.003	4*s*′ ^3^D_2_^o^	4*p*′ ^3^P_2_
2 855.3126	35 012.152 (0.002)	−0.003	4*s*′ ^3^D_2_^o^	4*p*′ ^3^P_1_
2 853.3087	35 036.731 (0.003)	0.006	4*s*′ ^3^D_1_^o^	4*p*′ ^3^P_1_
2 842.9654	35 164.199 (0.005)	0.003	4*s*′ ^3^D_1_^o^	4*p*′ ^3^P_0_
2 824.6461	35 392.250 (0.026)	−0.001	4*s*″ ^3^P_1_^o^	4*p*″ ^3^P_0_
2 818.2261	35 472.870 (0.035)	*	4*s*″ ^3^P_0_^o^	4*p*″ ^3^P_1_
2 796.6340	35 746.695 (0.005)	0.004	3*d*″ ^1^F_3_^o^	4*p*′ ^1^ D_2_
2 783.6035	35 914.061 (0.019)	−0.000	4*s*″ ^3^P_2_^o^	4*p*″ ^3^P_1_
2 762.1650	36 192.796 (0.006)	−0.003	4*s*″ ^3^P_2_^o^	4*p*″ ^3^P_2_
2 753.9120	36 301.217 (0.005)	−0.003	4*s*′ ^1^D_2_^o^	4*p*′ ^1^D_2_
2 724.7878	36 689.240 (0.004)	−0.000	3*d*′ ^3^D_3_^o^	4*p*′ ^3^D_3_
2 685.5827	37 224.825 (0.009)	−0.012	4*p*′ ^3^P^0^	4*d*′ ^3^D_1_^o^
2 678.3543	37 325.260 (0.005)	0.010	3*d*′ ^3^D_2_^o^	4*p*′ ^3^D_2_
2 673.9635	37 386.560 (0.005)	−0.003	4*p*′ ^3^P_1_	4*d*′ ^3^D_2_^o^
2 654.5492	37 659.972 (0.004)	−0.002	4*p*′ ^3^P_2_	4*d*′ ^3^D_3_^o^
2 631.8635	37 984.573 (0.012)	−0.007	3*d*′ ^3^D_1_^o^	4*p*′ ^3^D_1_
2 617.26 D	38 196.25 (0.22)	0.25	3*d*′ ^3^D_1_^o^	4*p*″ ^3^P_0_
2 602.12 D	38 418.74 (0.22)	−0.02	3*d*′ ^3^D_1_^o^	4*p*″ ^3^P_1_
2 591.4982	38 576.192 (0.005)	−0.013	4*p*″ ^3^P_2_	4*d*″ ^3^P_1_^o^
2 584.8765	38 674.998 (0.011)	−0.003	4*p*″ ^3^P_2_	4*d*″ ^3^P_2_^o^
2 583.39 D	38 697.47 (0.22)	−0.23	3*d*′ ^3^D_1_^o^	4*p*″ ^3^P_2_
2 579.6397	38 753.501 (0.001)	−0.000	4*s*″ ^1^P_1_^o^	3*p*^6 1^S_0_
2 566.3790	38 953.733 (0.018)	−0.001	4*p*″ ^3^P_1_	4*d*″ ^3^P_2_^o^
2 506.6580	39 881.750 (0.003)	−0.010	4*p*′ ^3^P_1_	4*d*′ ^3^P_2_^o^
2 504.3879	39 917.877 (0.007)	0.012	4*p*′ ^3^P^0^	4*d*′ ^3^P_1_^o^
2 494.8516	40 070.460 (0.011)	*	4*p*′ ^3^P_1_	4*d*′ ^3^P_0_^o^
2 488.8577	40 166.952 (0.002)	0.002	4*p*′ ^3^P_2_	4*d*′ ^3^P_2_^o^
2 484.1046	40 243.804 (0.012)	0.001	3*d*″ ^3^D_4_^o^	4*p*′ ^3^F_4_
2 479.76 D	40 314.45 (0.24)	−0.14	4*p*′ ^3^P^0^	4*d*′ ^3^ S_1_
2 478.7619	40 330.548 (0.006)	−0.008	4*p*′ ^3^P_2_	4*d*′ ^3^P_1_^o^
2 476.5289	40 366.908 (0.003)	−0.005	4*p*′ ^3^D_2_	4*d*′ ^3^D_2_^o^
2 476.057D	40 374.590 (0.008)	0.001	4*p*′ ^3^D^3^	4*d*′ ^3^D_3_^o^
2 472.9445	40 425.406 (0.002)	0.002	4*p*′ ^3^D^4^	4*d*′ ^3^D_4_^o^
2 471.92 D	40 441.92 (0.24)	0.24	4*p*′ ^3^P_1_	4*d*′ ^3^D_1_^o^
2 468.6732	40 495.345 (0.004)	0.002	4*p*″ ^3^P_2_	4*d*″ ^3^D_3_^o^
2 454.63 D	40 727.12 (0.24)	−0.11	4*p*′ ^3^P_2_	4*d*′ ^3^D_1_^o^
2 443.6241	40 910.412 (0.003)	0.013	4*p*′ ^3^P_2_	5*s*′ ^3^D_3_^o^
2 441.2307	40 950.532 (0.051)	*	4*p*″ ^3^P_1_	4*d*″ ^3^D_2_^o^
2 427.4901	41 182.325 (0.005)	−0.013	4*p*″ ^3^D^3^	4*d*″ ^3^D_3_^o^
2 426.1764	41 204.609 (0.007)	−0.000	4*p*″ ^3^D_2_	4*d*″ ^3^D_2_^o^
2 425.4917	41 216.238 (0.002)	0.002	4*p*″ ^3^D_2_	4*d*″ ^3^D_3_^o^
2 424.2959	41 236.571 (0.002)	−0.002	4*p* ^5^P^3^	4*d* ^5^D_3_^o^
2 423.9600	41 242.282 (0.001)	*	4*p* ^5^P^3^	4*d* ^5^D_4_^o^
2 423.5239	41 249.702 (0.003)	*	4*p*′ ^3^D^4^	4*d*′ ^3^D^o 5^
2 419.9242	41 311.058 (0.003)	−0.000	4*p*″ ^3^D_1_	4*d*″ ^3^D_2_^o^
2 418.8421	41 329.538 (0.003)	*	4*p*′ ^3^D^3^	4*d*′ ^3^D_4_^o^
2 416.0030	41 378.092 (0.004)	0.010	4*p* ^5^P_2_	4*d* ^5^D_1_^o^
2 415.8638	41 380.487 (0.002)	−0.001	4*p* ^5^P_2_	4*d* ^5^D_2_^o^
2 415.6512	41 384.126 (0.002)	0.001	4*p* ^5^P_2_	4*d* ^5^D_3_^o^
2 413.2214	41 425.793 (0.003)	−0.000	4*p*′ ^3^D_2_	4*d*′ ^3^D_3_^o^
2 411.0017	41 463.929 (0.002)	−0.000	4*p* ^5^P_1_	4*d* ^5^D_1_^o^
2 410.8622	41 466.324 (0.003)	0.002	4*p* ^5^P_1_	4*d* ^5^D_2_^o^
2 410.3798	41 474.639 (0.009)	−0.014	4*p*′ ^3^D^3^	4*d*′ ^3^D_3_^o^
2 405.0036	41 567.328 (0.002)	0.004	4*p*′ ^3^D_1_	4*d*′ ^3^D_2_^o^
2 404.5772	41 574.709 (0.003)	−0.007	4*p*′ ^3^D_2_	4*d*′ ^3^D_2_^o^
2 399.1952	41 667.972 (0.003)	−0.014	4*p*′ ^3^D^3^	4*d*′ ^3^D_4_^o^
2 395.6539	41 729.542 (0.008)	0.005	4*p*′ ^3^D_2_	4*d*′ ^3^D_3_^o^
2 377.7480	42 043.776 (0.003)	−0.061	4*s*′ ^1^D_2_^o^	4*p*″ ^1^P_1_
2 358.9539	42 378.691 (0.005)	0.022	4*p*′ ^3^D^3^	4*d*′ ^3^D_3_^o^
2 347.5774	42 584.065 (0.004)	−0.001	4*p*″ ^3^P_2_	5*s*″ ^3^P_2_^o^
2 345.1967	42 627.291 (0.003)	−0.000	3*d*′ ^3^D_3_^o^	4*p*′ ^3^P_2_
2 338.1614	42 755.541 (0.010)	*	4*p*″ ^3^D_1_	4*d*″ ^3^P_0_^o^
2 335.2989	42 807.944 (0.003)	−0.000	4*p*″ ^3^D_1_	4*d*″ ^3^P_1_^o^
2 329.9196	42 906.749 (0.012)	0.022	4*p*″ ^3^D_1_	4*d*″ ^3^P_2_^o^
2 319.2632	43 103.910 (0.004)	−0.012	4*p* ^3^P_2_	4*d* ^3^D_2_^o^
2 319.0123	43 108.564 (0.005)	−0.002	4*p* ^3^P^0^	4*d* ^3^D_1_^o^
2 317.9324	43 128.641 (0.002)	0.002	4*p* ^3^P_1_	4*d* ^3^D_2_^o^
2 317.3757	43 139.005 (0.003)	*	4*p* ^3^P_2_	4*d* ^3^D_3_^o^
2 316.8911	43 148.020 (0.005)	0.006	4*p* ^3^P_1_	4*d* ^3^D_1_^o^
2 312.1238	43 236.984 (0.011)	*	3*d*″ ^3^P_1_^o^	4*p*′ ^3^P_0_
2 300.7825	43 450.094 (0.031)	*	4*p* ^3^P_1_	5*s* ^3^D_1_^o^
2 292.9752	43 598.024 (0.002)	−0.000	4*p*′ ^3^D^3^	4*d*′ ^3^D_3_^o^
2 292.2647	43 611.527 (0.031)	0.007	4*s*′ ^3^D_3_^o^	4*p*″ ^3^D_3_
2 291.3594	43 628.768 (0.004)	−0.004	4*p*″ ^3^D^3^	4*d*″ ^3^D_3_^o^
2 282.2226	43 803.417 (0.004)	−0.001	3*d*′ ^3^D_2_^o^	4*p*′ ^3^P_1_
2 281.1989	43 823.070 (0.008)	0.001	4*p*′ ^3^D_1_	4*d*′ ^3^D_1_^o^
2 279.0328	43 864.717 (0.005)	−0.000	4*p*′ ^3^D_2_	4*d*′ ^3^D_2_^o^
2 269.9537	44 040.146 (0.001)	*	3*d*″ ^1^D_3_^o^	4*p*″ ^1^D_2_
2 269.7517	44 044.065 (0.002)	−0.000	4*s*″ ^1^P_1_^o^	4*p*″ ^1^S_0_
2 265.1420	44 133.641 (0.008)	−0.000	3*d*″ ^1^D_2_^o^	4*p*′ ^1^P_1_
2 242.3207	44 582.817 (0.011)	0.001	3*d*′ ^3^D_1_^o^	4*p*′ ^3^P_0_
2 241.7248	44 594.668 (0.002)	−0.000	4*s*′ ^1^D_2_^o^	4*p*″ ^1^D_2_
2 192.0137	45 605.898 (0.003)	−0.001	4*p*′ ^3^D^4^	5*s*′ ^3^D_3_^o^
2 191.1210	45 624.475 (0.072)	*	4*p*″ ^3^D_2_	5*s*″ ^3^P_1_^o^
2 188.1716	45 685.966 (0.004)	−0.000	4*p*′ ^3^D^3^	5*s*′ ^3^D_2_^o^
2 186.6626	45 717.489 (0.003)	−0.000	4*p*″ ^3^D^3^	5*s*″ ^3^P_2_^o^
2 184.0268	45 772.659 (0.006)	*	4*p*′ ^3^D_2_	5*s*′ ^3^D_1_^o^
2 177.1971	45 916.235 (0.018)	−0.006	4*p* ^5^P^3^	5*s* ^5^D_2_^o^
2 170.2217	46 063.789 (0.018)	0.005	4*p* ^5^P_2_	5*s* ^5^D_2_^o^
2 168.2822	46 105.004 (0.004)	−0.012	4*p*′ ^3^D^3^	4*d*′ ^3^P_2_^o^
2 148.3890	46 531.847 (0.032)	0.012	4*s*′ ^3^D_2_^o^	4*p*″ ^3^P_1_
2 135.3565	46 815.817 (0.005)	0.003	4*p*″ ^3^D_1_	5*s*″ ^3^P_2_^o^
2 133.8685	46 848.464 (0.003)	−0.001	4*p*′ ^3^D^3^	5*s*′ ^3^D_3_^o^
2 128.207D	46 973.080 (0.006)	−0.008	4*p*′ ^3^D_1_	5*s*′ ^3^D_1_^o^
2 125.1373	47 040.919 (0.004)	−0.000	4*p*′ ^3^D_2_	5*s*′ ^3^D_2_^o^
2 006.8504	49 813.208 (0.036)	*	3*d*″ ^3^P_2_^o^	4*p*″ ^3^S_1_
1 973.7936	50 663.849 (0.024)	0.009	4*s* ^3^D_1_^o^	4*p*′ ^3^P_2_
1 962.7446	50 949.051 (0.024)	0.014	4*s* ^3^D_1_^o^	4*p*′ ^3^P_1_
1 957.8466	51 076.519 (0.024)	0.006	4*s* ^3^D_1_^o^	4*p*′ ^3^P_0_
1 938.7892	51 578.584 (0.005)	*	3*d*′ ^1^D_4_^o^	4*p*′ ^1^F_3_
1 919.5200	52 096.342 (0.018)	0.016	3*d* ^3^D_1_^o^	4*p* ^3^P_1_
1 918.0681	52 135.798 (0.018)	−0.007	3*d* ^3^D_1_^o^	4*p* ^3^P_0_
1 915.5767	52 203.594 (0.016)	0.005	3*d* ^3^D_2_^o^	4*p* ^3^P_1_
1 914.6703	52 228.325 (0.016)	−0.013	3*d* ^3^D_2_^o^	4*p* ^3^P_2_
1 914.4119	52 235.365 (0.018)	−0.001	3*d* ^3^D_3_^o^	4*p* ^3^P_2_
1 878.0056	53 247.972 (0.072)	−0.006	3*d*″ ^3^D_2_^o^	4*p*″ ^3^D_1_
1 865.6622	53 600.271 (0.029)	*	3*d*″ ^3^D_3_^o^	4*p*″ ^3^D_2_
1 855.6497	53 889.499 (0.031)	−0.018	3*d*″ ^3^D_4_^o^	4*p*″ ^3^D_3_
1 843.0838	54 256.894 (0.090)	*	3*d*′ ^3^D_3_^o^	4*p*′ ^3^F_2_
1 839.3986	54 367.595 (0.059)	*	3*d*′ ^3^D_4_^o^	4*p*′ ^3^F_3_
1 836.3722	54 455.191 (0.039)	*	3*d*′ ^3^D^o 5^	4*p*′ ^3^F_4_
1 675.6232	59 679.270 (0.035)	0.018	3*d* ^5^D_2_^o^	4*p* ^5^P_1_
1 675.4773	59 684.485 (0.043)	−0.000	3*d* ^5^D_1_^o^	4*p* ^5^P_1_
1 673.4061	59 758.269 (0.043)	0.089	3*d* ^5^D_3_^o^	4*p* ^5^P_2_
1 673.2169	59 765.107 (0.035)	0.008	3*d* ^5^D_2_^o^	4*p* ^5^P_2_
1 673.0711	59 770.322 (0.043)	0.001	3*d* ^5^D_1_^o^	4*p* ^5^P_2_
1 669.6701	59 892.071 (0.061)	*	3*d* ^5^D_4_^o^	4*p* ^5^P_3_
1 669.2893	59 905.823 (0.043)	−0.089	3*d* ^5^D_3_^o^	4*p* ^5^P_3_
1 669.0970	59 912.661 (0.035)	−0.026	3*d* ^5^D_2_^o^	4*p* ^5^P_3_
1 617.7766	61 813.23 (0.29)	*	3*d*′ ^1^D_0_^o^	4*p*′ ^1^P_1_
1 614.7997	61 927.103 (0.058)	0.082	3*d*′ ^3^D_4_^o^	4*p*′ ^3^D_3_
1 611.0049	62 073.053 (0.071)	0.032	3*d*′ ^3^D_3_^o^	4*p*′ ^3^D_2_
1 586.6206	63 027.153 (0.058)	−0.113	3*d*′ ^3^D_4_^o^	4*p*′ ^3^F_3_
1 583.0377	63 169.668 (0.058)	0.022	3*d*′ ^3^D_4_^o^	4*p*′ ^3^F_4_
1 576.5915	63 428.006 (0.071)	−0.035	3*d*′ ^3^D_3_^o^	4*p*′ ^3^F_3_
1 572.3340	63 599.72 (0.10)	−0.000	3*d*′ ^3^D_2_^o^	4*p*′ ^3^F_2_
1 467.8533	68 126.780 (0.030)	−0.080	3*d* ^3^D_1_^o^	4*p*′ ^3^D_1_
1 465.7036	68 226.652 (0.028)	−0.034	3*d* ^3^D_2_^o^	4*p*′ ^3^D_2_
1 465.5506	68 233.692 (0.029)	0.049	3*d* ^3^D_3_^o^	4*p*′ ^3^D_2_
1 460.2487	68 481.555 (0.028)	−0.070	3*d* ^3^D_2_^o^	4*p*′ ^3^D_3_
1 460.0973	68 488.595 (0.029)	−0.009	3*d* ^3^D_3_^o^	4*p*′ ^3^D_3_
1 255.6374	79 640.83 (0.47)	*	3*p*^5 1^P_1_^o^	4*p*′ ^1^P_1_
887.4040	112 688.26 (0.13)	0.01	3*p*^4 3^P_1_	3*p*^5 3^ P_2_
883.1800	113 227.13 (0.23)	0.02	3*p*^4 3^P^0^	3*p*^5 3^ P_1_
879.6229	113 685.15 (0.13)	0.07	3*p*^4 3^P_1_	3*p*^5 3^ P_1_
878.7308	113 800.48 (0.13)	−0.13	3*p*^4 3^P_2_	3*p*^5 3^ P_2_
875.5354	114 215.82 (0.13)	*	3*p*^4 3^P_1_	3*p*^5 3^ P_0_
871.0995	114 797.37 (0.13)	−0.07	3*p*^4 3^P_2_	3*p*^5 3^P_1_^o^
695.5390	143 773.34 (0.32)	−0.05	3*p*^4 3^P_1_	3*d* ^5^D_1_^o^
643.2572	155 459.02 (0.43)	−0.21	3*p*^4 3^P^0^	3*d* ^3^D_1_^o^
641.8072	155 810.34 (0.09)	0.32	3*p*^4 3^P_1_	3*d* ^3^D_2_^o^
641.3658	155 917.60 (0.09)	0.34	3*p*^4 3^P_1_	3*d* ^3^D_1_^o^
637.2881	156 915.53 (0.08)	−0.63	3*p*^4 3^P_2_	3*d* ^3^D_3_^o^
636.8194	157 029.82 (0.08)	0.12	3*p*^4 3^P_2_	3*d* ^3^D_1_^o^
604.1590	165 519.51 (0.17)	0.17	3*p*^4 1^D_2_	3*d*″ ^1^D_2_^o^
578.3865	172 894.04 (0.11)	−0.72	3*p*^4 1^D_2_	3*d*′ ^3^D_2_^o^
577.1457	173 266.37 (0.32)	0.10	3*p*^4 3^P_1_	4*s* ^5^D_2_^o^
573.4666	174 378.59 (0.32)	−0.21	3*p*^4 3^P_2_	4*s* ^5^D_2_^o^
558.3231	179 107.53 (0.43)	0.23	3*p*^4 3^P^0^	4*s* ^3^D_1_^o^
556.8979	179 566.10 (0.08)	−0.05	3*p*^4 3^P_1_	4*s* ^3^D_1_^o^
553.4696	180 678.33 (0.08)	0.05	3*p*^4 3^P_2_	4*s* ^3^D_1_^o^
538.7890	185 601.23 (0.43)	0.18	3*p*^4 3^P^0^	3*d*′ ^3^D_1_^o^
537.4622	186 059.81 (0.09)	0.21	3*p*^4 3^P_1_	3*d*′ ^3^D_1_^o^
536.7451	186 307.93 (0.17)	−0.24	3*p*^4 1^D_2_	3*d*″ ^1^D_3_^o^
535.5881	186 711.74 (0.09)	1.09	3*p*^4 3^P_1_	3*d*′ ^3^D_2_^o^
529.9005	188 714.88 (0.08)	−0.21	3*p*^4 3^P_2_	3*d*′ ^3^D_3_^o^
511.5675	195 478.42 (0.09)	0.79	3*p*^4 3^P_1_	4*s*′ ^3^D_1_^o^
511.5018	195 503.00 (0.09)	0.27	3*p*^4 3^P_1_	4*s*′ ^3^D_2_^o^
508.4390	196 680.86 (0.08)	−0.43	3*p*^4 3^P_2_	4*s*′ ^3^D_3_^o^

KL This magnetic-dipole wavelength is from D. Kelly and J. H. Lacy, Astrophys. J. Lett. **454**, L161 (1995).

B These magnetic-dipole and electric-quadrupole wavelengths are from I. S. Bowen, Astrophys. J. **121**, 306 (1955).

D These wavelengths are taken from T. L. de Bruin, Proc. Roy. Acad. Amsterdam **40**, 340 (1937).

B2 This magnetic-dipole wavelength is from I. S. Bowen, Astrophys. J. **132**, 1 (1960).

* This is the the only transition connecting a level to the network; hence calculated and observed wavenumber are equal.

**Table 2:** Energy level values of Ar III and their standard uncertainties (in cm^−1^). The last column shows the number of transitions in Table 1 that involve this level

Configuration	Designation	*J*	Energy level value (cm^−1^)	Standard uncertainty (cm^−1^)	*n*
3*s*^2^3*p*^4^	3*p*^4 3^P	2	0.000	0.000	10
		1	1 112.175	0.006	15
		0	1 570.229	0.150	4
3*s*^2^3*p*^4^	3*p*^4 1^D	2	14 010.004	0.029	6
3*s*^2^3*p*^4^	3*p*^4 1^S	0	33 265.724	0.153	2
3*s*3*p*^5^	3*p*^5 3^P^o^	2	113 800.459	0.092	2
		1	114 797.353	0.092	3
		0	115 328.002	0.130	1
3*s*3*p*^5^	3*p*^5 1^P^o^	1	144 022.323^a^	0.485	1
3*s*^2^3*p*^3^(^4^S^o^)3*d*	3*d* ^5^D^o^	0			
		1	144 885.468	0.251	3
		2	144 890.681	0.254	3
		3	144 897.519	0.255	3
		4	144 911.271	0.259	1
3*s*^2^3*p*^3^(^4^S^o^)3*d*	3*d* ^3^D^o^	3	156 915.518	0.078	4
		2	156 922.558	0.078	5
		1	157 029.811	0.079	5
3*s*^2^3*p*^3^(^2^D^o^)3*d*	3*d*′ ^1^S^o^	0	161 849.923	0.323	1
3*s*^2^3*p*^3^(^2^D^o^)3*d*	3*d*′ ^3^F^o^	2	162 757.293	0.128	3
		3	163 076.157	0.105	2
		4	163 477.010	0.098	3
3*s*^2^3*p*^3^(^2^D^o^)3*d*	3*d*′ ^3^G^o^	3	172 100.118	0.118	1
		4	172 136.569	0.098	1
		5	172 191.487	0.088	1
3*s*^2^3*p*^3^(^4^S^o^)4*s*	4*s* ^5^S^o^	2	174 378.498	0.251	4
3*s*^2^3*p*^3^(^2^D^o^)3*d*	3*d*′ ^1^G^o^	4	175 665.583	0.159	1
3*s*^2^3*p*^3^(^2^P^o^)3*d*	3*d*″ ^1^D^o^	2	179 529.511	0.159	2
3*s*^2^3*p*^3^(^4^S^o^)4*s*	4*s* ^3^S^o^	1	180 678.316	0.076	8
3*s*^2^3*p*^3^(^2^P^o^)3*d*	3*d*″ ^3^F^o^	4	186 402.875	0.081	2
		3	186 658.191	0.111	1
		2	186 904.041	0.109	2
3*s*^2^3*p*^3^(^2^D^o^)3*d*	3*d*′ ^3^D^o^	1	187 172.018^a^	0.080	4
		2	187 823.950^a^	0.080	3
		3	188 714.874^a^	0.080	3
3*s*^2^3*p*^3^(^2^P^o^)3*d*	3*d*″ ^3^P^o^	0			
		1	188 517.851	0.080	2
		2	189 380.840	0.091	4
3*s*^2^3*p*^3^(^2^D^o^)4*s*	4*s*′ ^3^D^o^	1	196 590.636	0.080	5
		2	196 615.215	0.080	7
		3	196 680.847	0.080	6
3*s*^2^3*p*^3^(^2^D^o^)4*s*	4*s*′ ^1^D^o^	2	199 763.411	0.159	5
3*s*^2^3*p*^3^(^2^P^o^)3*d*	3*d*″ ^1^F^o^	3	200 317.932	0.159	3
3*s*^2^3*p*^3^(^4^S^o^)4*p*	4*p* ^5^P	1	204 569.951	0.251	5
		2	204 655.788	0.251	8
		3	204 803.342	0.251	7
3*s*^2^3*p*^3^(^2^D^o^)3*d*	3*d*′ ^3^S^o^	1	204 728.324	0.233	3
3*s*^2^3*p*^3^(^2^P^o^)4*s*	4*s*″ ^3^P^o^	2	207 233.004	0.084	3
		1	207 532.328	0.084	2
		0	207 674.194	0.091	1
3*s*^2^3*p*^3^(^4^S^o^)4*p*	4*p* ^3^P	1	209 126.152	0.077	6
		2	209 150.883	0.077	5
		0	209 165.608	0.077	3
3*s*^2^3*p*^3^(^2^P^o^)3*d*	3*d*″ ^3^D^o^	3	210 213.087	0.162	3
		2	211 005.831	0.146	4
		1	211 565.045	0.151	3
3*s*^2^3*p*^3^(^2^P^o^)4*s*	4*s*″ ^1^P^o^	1	211 063.766	0.159	4
3*s*^2^3*p*^3^(^2^D^o^)3*d*	3*d*′ ^3^P^o^	2	213 951.776	0.084	3
		1	214 347.609	0.084	2
		0	214 569.734	0.140	2
3*s*^2^3*p*^3^(^2^D^o^)3*d*	3*d*′ ^1^P^o^	1	219 908.474	0.159	2
3*s*^2^3*p*^3^(^2^D^o^)4*p*	4*p*′ ^1^P	1	223 663.153	0.159	4
3*s*^2^3*p*^3^(^2^D^o^)4*p*	4*p*′ ^3^D	2	225 149.210	0.080	10
		1	225 156.591	0.080	6
		3	225 404.113	0.080	11
3*s*^2^3*p*^3^(^2^D^o^)4*p*	4*p*′ ^3^F	2	226 357.012	0.080	6
		3	226 504.163	0.080	7
		4	226 646.678	0.080	7
3*s*^2^3*p*^3^(^2^D^o^)4*p*	4*p*′ ^1^F	3	227 244.168	0.159	2
3*s*^2^3*p*^3^(^2^D^o^)4*p*	4*p*′ ^3^P	2	231 342.165	0.080	9
		1	231 627.367	0.080	8
		0	231 754.835	0.080	7
3*s*^2^3*p*^3^(^2^D^o^)4*p*	4*p*′ ^1^D	2	236 064.627	0.159	2
3*s*^2^3*p*^3^(^2^P^o^)4*p*	4*p*″ ^3^S	1	239 194.048	0.084	8
3*s*^2^3*p*^3^(^2^P^o^)4*p*	4*p*″ ^3^D	1	240 152.013	0.085	4
		2	240 258.462	0.084	9
		3	240 292.376	0.084	9
3*s*^2^3*p*^3^(^2^P^o^)4*p*	4*p*″ ^1^P	1	241 807.185	0.159	2
3*s*^2^3*p*^3^(^2^P^o^)4*p*	4*p*″ ^3^P	0	242 924.579	0.088	3
		1	243 147.064	0.085	7
		2	243 425.799	0.084	9
3*s*^2^3*p*^3^(^2^P^o^)4*p*	4*p*″ ^1^D	2	244 358.078	0.159	2
3*s*^2^3*p*^3^(^4^S^o^)4*d*	4*d* ^5^D^o^	0			
		1	246 033.879	0.251	2
		2	246 036.275	0.251	2
		3	246 039.914	0.251	2
		4	246 045.624	0.251	1
3*p*^6^	3*p*^6 1^S	0	249 817.267	0.159	2
3*s*^2^3*p*^3^(^4^S^o^)5*s*	5*s* ^5^S^o^	2	250 719.577	0.252	2
3*s*^2^3*p*^3^(^4^S^o^)4*d*	4*d* ^3^D^o^	2	252 254.794	0.077	2
		1	252 274.172	0.077	2
		3	252 289.888	0.077	1
3*s*^2^3*p*^3^(^4^S^o^)5*s*	5*s* ^3^S^o^	1	252 576.246	0.082	1
3*s*^2^3*p*^3^(^2^P^o^)4*p*	4*p*″ ^1^S	0	255 107.831	0.159	1
3*s*^2^3*p*^3^(^2^D^o^)4*d*	4*d*′ ^3^F^o^	2	266 723.919	0.080	3
		3	266 878.753	0.080	2
		4	267 072.085	0.080	3
3*s*^2^3*p*^3^(^2^D^o^)4*d*	4*d*′ ^3^G^o^	3	267 782.804	0.080	2
		4	267 833.701	0.080	1
		5	267 896.381	0.080	1
3*s*^2^3*p*^3^(^2^D^o^)4*d*	4*d*′ ^3^D^o^	1	268 979.661	0.080	2
		3	269 002.137	0.080	2
		2	269 013.927	0.080	2
3*s*^2^3*p*^3^(^2^D^o^)4*d*	4*d*′ ^3^P^o^	2	271 509.117	0.080	3
		1	271 672.713	0.080	2
		0	271 697.827	0.080	1
3*s*^2^3*p*^3^(^2^D^o^)4*d*	4*d*′ ^3^S^o^	1	272 069.287	0.249	3
3*s*^2^3*p*^3^(^2^D^o^)5*s*	5*s*′ ^3^D^o^	1	272 129.671	0.080	1
		2	272 190.129	0.080	2
		3	272 252.577	0.080	3
3*s*^2^3*p*^3^(^2^P^o^)4*d*	4*d*″ ^3^F^o^	2	281 463.071	0.085	2
		3	281 474.701	0.084	2
		4			
3*s*^2^3*p*^3^(^2^P^o^)4*d*	4*d*″ ^3^P^o^	0	281 949.589	0.085	1
		1	282 001.992	0.084	2
		2	282 100.797	0.085	3
3*s*^2^3*p*^3^(^2^P^o^)4*d*	4*d*″ ^3^D^o^	3	283 921.144	0.084	2
		2	284 097.597	0.099	1
		1			
3*s*^2^3*p*^3^(^2^P^o^)5*s*	5*s*″ ^3^P^o^	2	286 009.865	0.084	3
		1	285 882.937	0.110	1
		0			

a This level is not named for its major component. See Table 4 for percentage compositions.

**Table 3:** Parameter values for the 3*s*3*p*^5^ + 3*s*^2^3*p*^3^3*d* + 3*s*^2^3*p*^3^4*d* energy fit in Ar III

Parameter	Fitted value and standard uncertainty^a^ (cm^−1^)	*HFR* value^b^ (cm^−1^)	Ratio *HFR*/fitted value
*E*_av_(*sp*^5^)	152 728 ± 469		
ζ 3*p*	1 010	1 010	1.00
*G*^1^(3*s*3*p*)	80 158 ± 1162	94 485	0.85
*E*_av_(*p*^3^3*d*)	180 316 ± 159		
*F*^2^(3*p*3*p*)	57 576 ± 479	69 716	0.83
ζ 3*p*	1 047	1 047	1.00
ζ 3*d*	26	26	1.00
*F*^2^(3*p*3*d*)	45 580 ± 529	54 962	0.83
*G*^1^(3*p*3*d*)	53 118 ± 658	68 640	0.77
*X*^2^(3*p*3*d*)^c^	4 595 ± 652		
*G*^3^(3*p*3*d*)	33 838 ± 663	41 139	0.82
*E*_av_(*p*^3^4*d*)	268 455 ± 154		
*F*^2^(3*p*3*p*)	58 419 ± 428	72 001	0.81
ζ 3*p*	1 111	1 111	1.00
ζ 4*d*	7	7	1.00
*F*^2^(3*p*4*d*)	8 067 ± 2020	11 700	0.69
*G*^1^(3*p*4*d*)	5 983 ± 608	8 964	0.67
*G*^3^(3*p*4*d*)	4 035 ± 410	6 046	0.67
*R*^1^(3*p*3*p*,3*s*3*d*)	64 267 ± 343	78 908	0.81
*R*^1^(3*p*3*p*,3*s*4*d*)	20 735 ± 1965	27 625	0.75
*R*^0^(3*p*3*d*,3*p*4*d*)	0	0	1.00
*R*^2^(3*p*3*d*,3*p*4*d*)	12 405 ± 1036	16 813	0.74
*R*^1^(3*p*3*d*,4*d*3*p*)	17 113 ± 1429	23 194	0.74
*R*^3^(3*p*3*d*,4*d*3*p*)	10 823 ± 904	14 669	0.74
Standard deviation of the fit = 363 cm^−1^			

a Where no standard uncertainty is given, the parameter was held fixed at the value given.

b This value from the Cowan program is a relativistic approximation of the Hartree-Fock value.

c No ab initio value is available for *X*^2^.

**Table 4:** Percent composition of the 3*s*3*p*^5^, 3*s*^2^3*p*^3^3*d*, and 3*s*^2^3*p*^3^4*d* levels derived from a least-squares fit of the parameters to the levels of these three configurations

Energy level value (cm^−1^)	Percent composition		
*J*=0			
115 328.002	76 *sp*^5^(^2^S)^3^P	18 *p*^3^3*d*(^2^D)^3^P	6 *p*^3^3*d*(^2^P)^3^P
144 785.7^b^	100 *p*^3^3*d*(^4^S)^5^D		
161 849.923^a^	100 *p*^3^3*d*(^2^D)^1^S		
188 045.2^b^	77 *p*^3^3*d*(^2^P)^3^P	23 *p*^3^3*d*(^2^D)^3^P	
214 569.734	59 *p*^3^3*d*(^2^D)^3^P	23 *sp*^5^(^2^S)^3^P	16 *p*^3^3*d*(^2^P)^3^P
245 894.8^b^	100 *p*^3^4*d*(^4^S)^5^D		
266 922.3^b^	100 *p*^3^4*d*(^2^D)^1^S		
271 697.827	97 *p*^3^4*d*(^2^D)^3^P		
281 949.589	99 *p*^3^4*d*(^2^P)^3^P		
*J*=1			
114 797.353	76 *sp*^5^(^2^S)^3^P	17 *p*^3^3*d*(^2^D)^3^P	7 *p*^3^3*d*(^2^P)^3^P
144 022.323^c^	43 *sp*^5^(^2^S)^1^P	50 *p*^3^3*d*(^2^D)^1^P	6 *p*^3^3*d*(^2^P)^1^P
144 885.468	100 *p*^3^3*d*(^4^S)^5^D		
157 029.811	48 *p*^3^3*d*(^4^S)^3^D	47 *p*^3^3*d*(^2^D)^3^D	4 *p*^3^3*d*(^2^P)^3^D
187 172.018^c^	36 *p*^3^3*d*(^2^D)^3^D	50 *p*^3^3*d*(^2^P)^3^D	15 *p*^3^3*d*(^4^S)^3^D
188 517.851	78 *p*^3^3*d*(^2^P)^3^P	22 *p*^3^3*d*(^2^D)^3^P	
204 728.324^a^	99 *p*^3^3*d*(^2^D)^3^S		
211 565.045	44 *p*^3^3*d*(^2^P)^3^D	33 *p*^3^3*d*(^4^S)^3^D	15 *p*^3^3*d*(^2^D)^3^D
214 347.609	59 *p*^3^3*d*(^2^D)^3^P	22 *sp*^5^(^2^S)^3^P	15 *p*^3^3*d*(^2^P)^3^P
219 908.474	48 *p*^3^3*d*(^2^D)^1^P	37 *sp*^5^(^2^S)^1^P	11 *p*^3^3*d*(^2^P)^1^P
240 056.2^b^	75 *p*^3^3*d*(^2^P)^1^P	15 *sp*^5^(^2^S)^1^P	9 *p*^3^4*d*(^2^P)^1^P
246 033.879	100 *p*^3^4*d*(^4^S)^5^D		
252 274.172	88 *p*^3^4*d*(^4^S)^3^D	5 *p*^3^4*d*(^2^D)^3^D	
268 979.661	94 *p*^3^4*d*(^2^D)^3^D	3 *p*^3^4*d*(^4^S)^3^D	
271 199.1^b^	92 *p*^3^4*d*(^2^D)^1^P	4 *sp*^5^(^2^S)^1^P	
271 672.713	98 *p*^3^4*d*(^2^D)^3^S		
272 069.287	95 *p*^3^4*d*(^2^D)^3^P		
282 001.992	99 *p*^3^4*d*(^2^P)^3^P		
283 657.2^b^	97 *p*^3^4*d*(^2^P)^3^D		
292 098.8^b^	90 *p*^3^4*d*(^2^P)^1^P	8 *p*^3^3*d*(^2^P)^1^P	
*J*=2			
113 800.459	76 *sp*^5^(^2^S)^3^P	17 *p*^3^3*d*(^2^D)^3^P	7 *p*^3^3*d*(^2^P)^3^P
144 890.681	100 *p*^3^3*d*(^4^S)^5^D		
156 922.558	48 *p*^3^3*d*(^4^S)^3^D	47 *p*^3^3*d*(^2^D)^3^D	4 *p*^3^3*d*(^2^P)^3^D
162 757.293	84 *p*^3^3*d*(^2^D)^3^F	15 *p*^3^3*d*(^2^P)^3^F	
179 529.511	75 *p*^3^3*d*(^2^P)^1^D	25 *p*^3^3*d*(^2^D)^1^D	
186 904.041	84 *p*^3^3*d*(^2^P)^3^F	15 *p*^3^3*d*(^2^D)^3^F	
187 823.950^c^	35 *p*^3^3*d*(^2^D)^3^D	49 *p*^3^3*d*(^2^P)^3^D	15 *p*^3^3*d*(^4^S)^3^D
189 380.840	79 *p*^3^3*d*(^2^P)^3^P	21 *p*^3^3*d*(^2^D)^3^P	
211 005.831	44 *p*^3^3*d*(^2^P)^3^D	33 *p*^3^3*d*(^4^S)^3^D	15 *p*^3^3*d*(^2^D)^3^D
213 951.776	60 *p*^3^3*d*(^2^D)^3^P	22 *sp*^5^(^2^S)^3^P	14 *p*^3^3*d*(^2^P)^3^P
217 177.3^b^	72 *p*^3^3*d*(^2^D)^1^D	24 *p*^3^3*d*(^2^P)^1^D	3 *p*^3^4*d*(^2^D)^1^D
246 036.275	100 *p*^3^4*d*(^4^S)^5^D		
252 254.794	88 *p*^3^4*d*(^4^S)^3^D	5 *p*^3^4*d*(^2^D)^3^D	
266 723.919	97 *p*^3^4*d*(^2^D)^3^F		
269 013.927	94 *p*^3^4*d*(^2^D)^3^D	4 *p*^3^4*d*(^4^S)^3^D	
271 509.117	96 *p*^3^4*d*(^2^D)^3^P		
272 793.5^b^	86 *p*^3^4*d*(^2^D)^1^D	10 *p*^3^4*d*(^2^P)^1^D	
281 463.071	97 *p*^3^4*d*(^2^P)^3^F		
282 100.797	98 *p*^3^4*d*(^2^P)^3^P		
283 347.7^b^	44 *p*^3^4*d*(^2^P)^1^D	51 *p*^3^4*d*(^2^P)^3^D	3 *p*^3^4*d*(^2^D)^1^D
284 097.597	46 *p*^3^4*d*(^2^P)^3^D	44 *p*^3^4*d*(^2^P)^1^D	7 *p*^3^4*d*(^2^D)^1^D
*J*=3			
144 897.519	100 *p*^3^3*d*(^4^S)^5^D		
156 915.518	48 *p*^3^3*d*(^4^S)^3^D	48 *p*^3^3*d*(^2^D)^3^D	4 *p*^3^3*d*(^2^P)^3^D
163 076.157	85 *p*^3^3*d*(^2^D)^3^F	14 *p*^3^3*d*(^2^P)^3^F	
172 100.118	100 *p*^3^3*d*(^2^D)^3^G		
186 658.191	85 *p*^3^3*d*(^2^P)^3^F	14 *p*^3^3*d*(^2^D)^3^F	
188 714.874^c^	35 *p*^3^3*d*(^2^D)^3^D	49 *p*^3^3*d*(^2^P)^3^D	15 *p*^3^3*d*(^4^S)^3^D
200 317.932	63 *p*^3^3*d*(^2^P)^1^F	37 *p*^3^3*d*(^2^D)^1^F	
210 213.087	44 *p*^3^3*d*(^2^P)^3^D	33 *p*^3^3*d*(^4^S)^3^D	15 *p*^3^3*d*(^2^D)^3^D
225 295.2^b^	57 *p*^3^3*d*(^2^D)^1^F	35 *p*^3^3*d*(^2^P)^1^F	7 *p*^3^4*d*(^2^D)^1^F
246 039.914	100 *p*^3^4*d*(^4^S)^5^D		
252 289.888	89 *p*^3^4*d*(^4^S)^3^D	5 *p*^3^4*d*(^2^D)^3^D	
266 878.753	97 *p*^3^4*d*(^2^D)^3^F		
267 782.804	98 *p*^3^4*d*(^2^D)^3^G		
269 002.137	95 *p*^3^4*d*(^2^D)^3^D	4 *p*^3^4*d*(^4^S)^3^D	
275 804.9^b^	87 *p*^3^4*d*(^2^D)^1^F	7 *p*^3^4*d*(^2^P)^1^F	4 *p*^3^3*d*(^2^D)^1^F
281 474.701	97 *p*^3^4*d*(^2^P)^3^F		
283 921.144	97 *p*^3^4*d*(^2^P)^3^D		
286 173.7^b^	91 *p*^3^4*d*(^2^P)^1^F	5 *p*^3^4*d*(^2^D)^1^F	
*J*=4			
144 911.271	100 *p*^3^3*d*(^4^S)^5^D		
163 477.010	87 *p*^3^3*d*(^2^D)^3^F	13 *p*^3^3*d*(^2^P)^3^F	
172 136.569	100 *p*^3^3*d*(^2^D)^3^G		
175 665.583	100 *p*^3^3*d*(^2^D)^1^G		
186 402.875	86 *p*^3^3*d*(^2^P)^3^F	13 *p*^3^3*d*(^2^D)^3^F	
246 045.624	100 *p*^3^4*d*(^4^S)^5^D		
267 072.085	96 *p*^3^4*d*(^2^D)^3^F	3 *p*^3^4*d*(^2^D)^3^G	
267 833.701	96 *p*^3^4*d*(^2^D)^3^G	3 *p*^3^4*d*(^2^D)^3^F	
267 961.2^b^	99 *p*^3^4*d*(^2^D)^1^G		
281 629.2^b^	99 *p*^3^4*d*(^2^P)^3^F		
*J*=5			
172 191.487	100 *p*^3^3*d*(^2^D)^3^G		
267 896.381	100 *p*^3^4*d*(^2^D)^3^G		

a This known level was not used in the least-squares fit.

b The value of this level has not been determined experimentally. The value given is from the least-squares fit.

c This level is not named for its major eigenvector component.
